# Red blood cell distribution width as a predictor of survival in nasal-type, extranodal natural killer/T-cell lymphoma

**DOI:** 10.18632/oncotarget.21439

**Published:** 2017-09-30

**Authors:** Huaichao Luo, Xiaoying Quan, Xiao-Yu Song, Li Zhang, Yilin Yin, Qiao He, Shaolei Cai, Shi Li, Jian Zeng, Qing Zhang, Yu Gao, Sisi Yu

**Affiliations:** ^1^ Department of Clinical Laboratory, Sichuan Cancer Hospital & Institute, Sichuan Cancer Center, School of Medicine, University of Electronic Science and Technology of China, Chengdu, Sichuan, China; ^2^ Department of Medical Oncology, Sichuan Cancer Hospital & Institute, Sichuan Cancer Center, School of Medicine, University of Electronic Science and Technology of China, Chengdu, Sichuan, China; ^3^ Department of Biology, The Northeastern University, Huntington Ave, Boston, MA, USA; ^4^ Radiotherapy Center, Sichuan Cancer Hospital & Institute, Sichuan Cancer Center, School of Medicine, University of Electronic Science and Technology of China, Chengdu, Sichuan, China

**Keywords:** red blood cell distribution width (RDW), extranodal natural killer (NK)/T-cell lymphoma, predictor, survival, radiotherapy-based treatment

## Abstract

We retrospectively enrolled 191 nasal-type, extranodal natural killer/T-cell lymphoma (ENKTL) patients newly diagnosed from 2008 to 2016 at the Sichuan Cancer Hospital, in order to evaluate the relationship between disease outcomes, demographic and clinical factors, and red blood cell distribution width (RDW). C-index, fisher's exact test, univariate analysis, and cox regression analysis were applied. The median age of patients was 44 years and 134 (70%) were men. The cutoff of RDW was 46.2 fL determined by Cutoff Finder. Patients with RDW≤46.2 fL had significantly better progression-free survival (PFS) (3-year PFS, 80.4% vs. 63.1%; *P*=0.01) and overall survival (OS) (3-year OS, 83.2% vs. 65.5%; *P*=0.004) than those with RDW>46.2 fL. Multivariate analysis demonstrated that elevated RDW is an independent adverse predictor of OS (*P*=0.021, HR=2.04). RDW is an independent predictor of survival outcomes in ENKTL, which we found to be superior to both the prognostic index of natural killer lymphoma (PINK) and the Korean Prognostic Index (KPI) in discriminating patients with different outcomes in low-risk and high-risk groups (all *P* < 0.05). The new models combining RDW with the International Prognostic Index (IPI), KPI, and PINK showed more powerful prognostic value than corresponding original models. RDW represents an easily available and inexpensive marker for risk stratification in patients with ENKTL treated with radiotherapy-based treatment. Further prospective studies are warranted to confirm the prognostic value of RDW in ENKTL.

## INTRODUCTION

Nasal-type, extranodal natural killer/T-cell lymphoma (ENKTL) is a highly aggressive and heterogeneous non-Hodgkin lymphoma (NHL). It is most common in East Asia and Latin America (upwards of 10% of all NHLs), but rare in North America and Europe (less than 1% of all NHL) [1]. ENKTL accounts for 5–10% of all malignant lymphomas in China [2]. Depending on the primary site of the cancer, ENKTL is divided into upper aerodigestive tract NK/T-cell lymphoma (UNKTL) and extra-upper aerodigestive tract NK/T-cell lymphoma (EUNKTL). UNKTL typically causes the vast majority of cases of “lethal midline granuloma”, a syndrome which involves the paranasal sinuses, orbits, and lymph nodes, and even develops outside of the nasopharynx [1].

The red blood cell distribution width (RDW), which is traditionally known as anisocytosis, reflects the size heterogeneity of erythrocyte volume. RDW has been used to discriminate between different types of anemia [3]; however, emerging evidence suggests that anisocytosis is involved in a variety of human disorders such as cardiovascular disease, venous thromboembolism, diabetes, community-acquired pneumonia, as well as other acute or chronic conditions [3]. The role of RDW in patients with various cancers was investigated by several studies [4], and RDW has proven to be an independent prognosis factor in breast cancer [5], esophageal squamous cell carcinoma [6], lung cancer [7], and diffuse large B-cell lymphoma (DLBCL) [8]. However to the best of our knowledge the prognostic value of RDW in T-cell NHL has never been investigated. Therefore, we performed this study to evaluate the prognostic significance of RDW in patients with ENKL.

## RESULTS

### Patient characteristics

The main baseline characteristics of the 191 patients studied are listed in Table 1. The median age was 44 years (range = 15–86 years). Most of enrolled patients were male (134, 70.2%). There was an Ann-Arbor (AA) staging I/II predominance. In all, 184 patients (96.3%) were classified into AA I/II and 7 cases (3.7 %) into stage III/IV. The mean RDW was 45.7 (range = 36.3-65.0) (data not shown).

**Table 1:** Patient baseline characteristics by RDW Level

Characteristics	Total, n=191 (%)	RDW≤46.2 fL,n=107 (%)	RDW>46.2 fL,n=84 (%)	P
**Age(median [range], yr)**	44 (15,86)			0.078
≤60	159(83.2)	94(87.9)	65(77.4)	
>60	32(16.8)	13(12.1)	19(22.6)	
**Gender (male)**	134(70.2)	75(70.1)	59(70.2)	1.00
**ECOG PS**				**<0.001**
0∼1	157(82.2)	99(92.5)	58(69.0)	
≥2	34(17.8)	8(7.5)	26(31.0)	
**B symptoms (yes)**	88(46.1)	50(46.7)	38(45.2)	0.884
**LDH > 240 U/L**	49(25.7)	29(27.1)	20(23.8)	0.621
**Extranodal sites > 2**	80(41.9)	44(41.4)	36(42.9)	0.883
**Ki67 > 50%**	Available (161)	Available (91)	Available (70)	0.266
	91(56.6)	55(60.4)	36(51.4)	
**HB (≤126 g/L)**	84(43.9%)	45(42.1)	39(46.4)	0.560
**Ann Arbor stage**				1.00
I/II	184(96.3)	103(96.3)	81(96.3)	
III/IV	7(3.7)	4(3.7)	3(3.6)	
**Local invasiveness**	89(46.6)	50(46.7)	39(46.4)	1.00
**Regional lymphadenopathies**	80(41.9)	52(48.6)	28(33.3)	**0.039**
**Comorbidities**				
Smoking	51(26.7)	32(29.9)	19(22.6)	0.323
Drinking	28(14.7)	20(18.7)	8(9.50)	0.099
Hepatitis B	12(6.30)	7(6.50)	5(6.00)	1.00
Underlying diseases	28(14.7)	16(15.0)	12(14.3)	1.00
**IPI score**				**0.009**
0-1	139(72.8)	86(80.4)	53(63.1)	
2-5	52(27.2)	21(19.6)	31(36.9)	
**PINK score**				0.068
0	153(80.1)	91(85.0)	62(73.8)	
1-3	38(19.9)	16(15.0)	22(26.2)	
**KPI score**				0.219
0-1	127(66.5)	67(62.6)	60(71.4)	
2-4	64(33.5)	40(37.4)	24(28.6)	

NOTE. Regional lymphadenopathies defined as involvement of lymph nodes corresponding to N1-N3 of the primary lesion at the TNM staging system; local invasiveness defined as T3 or T4; Underlying diseases consisted of hypertension, hyperglycemia, hyperlipidemia and chronic bronchitis; Hepatitis B defined as serum HBsAg is positive. Characteristics with P values<0.05 are marked in bold. Abbreviations: ECOG PS, Eastern Cooperative Oncology Group performance status; HB, Hemoglobin; LDH, lactate dehydrogenase; EBER, EBV-encoded RNA; IPI, international prognostic index; KPI, Korean Prognostic Index; PINK, prognostic index of natural killer lymphoma.

**Figure 1:**
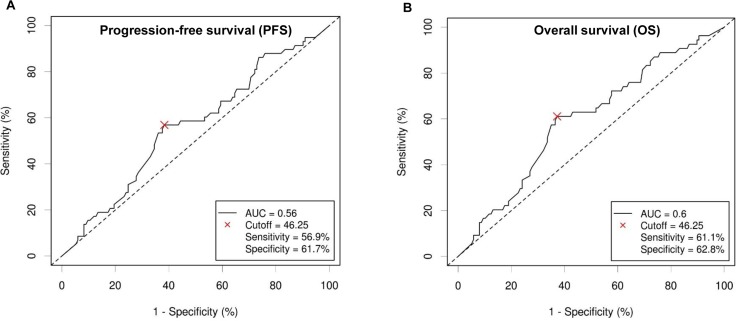
Receiver operating curve (ROC) from Cutoff Finder for determining the optimal cut-off value in predicting progression-free survival (PFS) **(A)** and overall survival (OS) **(B)** for red blood cell distribution width (RDW).

The optimal cut-off value of RDW for OS and PFS was determined by Cutoff Finder via the method of ROC curve (Euclidean distance). These two outcomes have the same cut-off value of 46.2 fL. For OS and PFS, the areas under the curve (AUC) were 0.60 and 0.56, sensitivities were 61.1 and 56.9, and specificities were 62.8 and 61.7, respectively (Figure 1). In all, 107 (56.0%) cases had low RDW (≤ 46.2 fL) and were classified into the low-risk group, whereas 84 (44%) patients had high RDW (>46.2 fL) and were classified into the high-risk group. The high-risk group tended to have a higher death ratio than that of the low-risk group (38.8% and 19.4%, respectively). Regional lymphadenopathies were more frequent in patients with low RDW than in other patients. Patients in the high-risk group tended to have ECOG PS ≥ 2, and IPI scores 2-5. Other characteristics showed no significant differences across high- or low-risk groups with different RDW levels (Table 1).

### Treatment modalities and response

Treatment modalities and responses are listed in Table 2. Of 191 patients, 40 (20.9%) received radiotherapy alone while 151 (79.1%) had chemotherapy combined with radiation. Of those 151 patients, 55 (28.7%) cases received chemotherapy followed by radiotherapy, 22 (11.5%) had concurrent chemoradiotherapy, 14 (7.3%) had concurrent chemoradiotherapy followed by chemotherapy, 45 (23.6%) had chemotherapy before and after radiotherapy, and 15 (7.8%) had radiotherapy followed by chemotherapy. The chemotherapy regimens included VDLP (4 patients), LVP (8 patients), P-Gemox (79 patients), CHOP or CHOP-like (60 patients).

**Table 2:** Treatment modalities and response in Patients with Extranodal Natural Killer (NK)/T-Cell Lymphoma

Treatment	RDW≤46.2 fL,n (%)	RDW>46.2 fL,n (%)	P
**Chemotherapy regimens**	86 (100.0)	65 (100.0)	**0.011**
CHOP or CHOP-like	36(41.9)	43(66.2)	
P-Gemox	46(53.5)	14(21.5)	
LVD/VDLP	4(4.6)	8(12.3)	
**Treatment outcome**	107(100.0)	84(100.0)	0.211
CR+PR	100(93.5)	74(88.1)	
SD+PD	7(6.5)	10(11.9)	
**Chemotherapy cycles**	86 (100.0)	65 (100.0)	0.191
<4	38(44.2)	26(55.4)	
≥4	48(55.8)	29(44.6)	
**Treatment modalities**	107(100.0)	84(100.0)	0.679
RT	21(19.6)	19(22.6)	
CT followed by RT	34(31.8)	21(25.0)	
Concurrent CRT	11(10.3)	11(13.1)	
Concurrent CRT followed by CT	8(7.5)	6(7.1)	
CT sandwiched RT	27(25.2)	18(21.4)	
RT followed by CT	6(5.6)	9(10.7)	

Abbreviations: CHOP, cyclophosphamide + doxorubicin + vincristine + prednisone; P-Gemox, pegaspargase + gemcitabine + oxaliplatin; LVD, l-asparaginase + vincristine + prednisone; VDLP, etoposide + cisplatin + l-asparaginase + dexamethasone; CR, complete response; PR, partial response; SD, stable disease; PD, progressive disease; CT, chemotherapy; RT, radiotherapy; CRT, Chemoradiotherapy.

Significant differences in chemotherapy regimens of patients was based on RDW (*P*=0.011); however, we found no significant associations between RDW and treatment response, chemotherapy cycles, and treatment modalities (Table 2).

### Survival and prognostic factors

**Figure 2:**
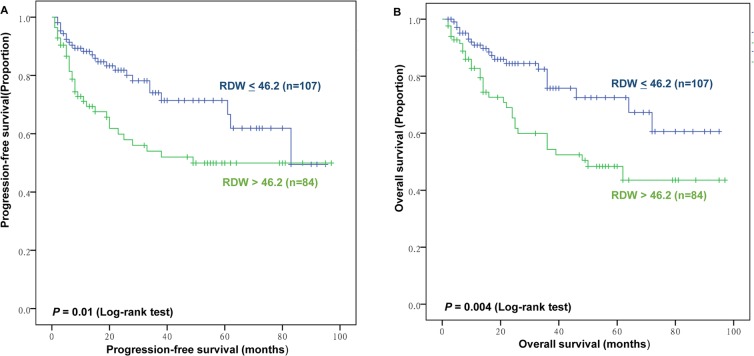
Prognostic value of red blood cell distribution width (RDW) for progression-free survival (PFS) and overall survival (OS) **(A)** PFS of patients according to RDW<46.2 fL versus RDW>46.2 fL. **(B)** OS of patients according to RDW <46.2 fL versus RDW>46.2 fL.

**Figure 3:**
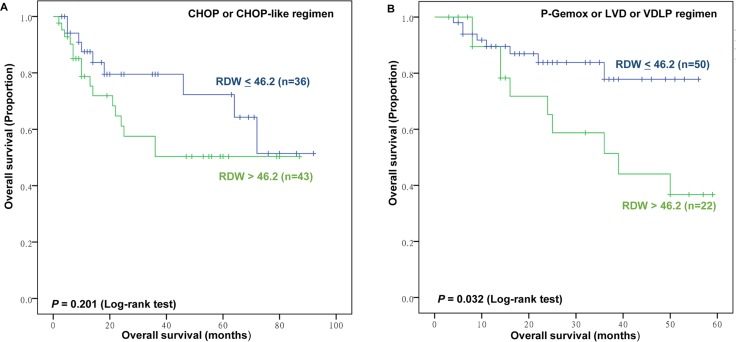
Subgroup analysis for prognostic value of red blood cell distribution width (RDW) for overall survival (OS) Kaplan–Meier plots for patients who received CHOP or CHOP-like **(A)** and non-CHOP or CHOP-like (P-Gemox or LVD or VDLP) **(B)** regimen according to RDW<46.2 fL versus RDW>46.2 fL.

Of the 191 patients studied, 54 patients died after a median follow-up of 30 months (range = 2–97). The 3-year OS and PFS in the whole group were 75.4% and 72.8%, respectively. The results of RDW distinguished two groups with significantly different survival outcomes (Figure 2). Patients with RDW≤46.2 fL had significantly better PFS than those with RDW>46.2 fL (3-year PFS, 80.4% vs. 63.1%; *P*=0.01, Figure 2A) and OS (3-year OS, 83.2% vs. 65.5%; *P*=0.004, Figure 2B). Due to the existence of significant differences in chemotherapy regimens of patients based on RDW, we conducted subgroup analysis. We discovered that RDW is a significant predictor of overall survival for patients treated with P-Gemox, LVD, or VDLP regimen, but not CHOP or CHOP-like regimen (P=0.032, P=0.201, respectively, Figure 3). Nevertheless, patients harboring RDW>46.2 fL tended to have worse OS than those with RDW≤46.2 fL (3-year OS, 62.8% vs. 83.3%; mean OS, 51.9 months vs. 67.2 months). Thus, we still consider RDW a weak predictive index for patients with CHOP or CHOP-like regimen.

The results of univariate and multivariate analysis are displayed in Table 3. In the multivariate analysis for PFS, RDW >46.2 fL, HB>126 g/L, and local invasiveness were found to be significant independent predictors of PFS (*P*=0.03, HR=1.895; *P*=0.036, HR=0.555; *P*=0.023, HR=2.204, respectively). Furthermore, RDW>46.2 fL, HB>126 g/L, and local invasiveness remained significantly predictive of OS (*P*=0.021, HR=2.04; *P*=0.016, HR=0.49; *P*=0.03, HR=2.244, respectively) in the multivariate analysis for OS.

**Table 3:** Univariate and Multivariate Analysis of Prognostic Factors for PFS and OS in Patients with ENKL

	PFS			OS		
Factors	Univariate analysis	Multivariate analysis		Univariate analysis	Multivariate analysis	
	*P*	HR(95%CI)	*P*	*P*	HR(95%CI)	*P*
**Age >60 years**	0.299			0.145		
**Gender, male**	0.608			0.763		
**ECOG PS >2**	**<0.001**			**<0.001**		
**B symptoms, yes**	0.877			0.92		
**LDH > 240 U/L**	**0.03**			**0.026**		
**Extranodal sites > 2**	**0.001**			**0.001**		
**Ki67 > 50%**	0.16			0.154		
**Ann Arbor stage, III/IV**	0.071			0.122		
**Local invasiveness**	**0.004**	**2.204(1.117-4.349)**	**0.023**	**0.009**	**2.244(1.08-4.663)**	**0.03**
**Regional lymphadenopathies**	0.078			0.117		
**Smoking**	0.063			0.15		
**Drinking**	0.232			0.876		
Hepatitis B	0.511			0.605		
**HB>126g/L**	**0.001**	**0.555(0.320-0.963)**	**0.036**	**<0.001**	**0.490(0.275-0.874)**	**0.016**
**Underlying diseases**	**0.007**			**0.002**		
**IPI score >2**	**<0.001**			**<0.001**		
**PINK score >1**	**0.042**			**0.019**		
**KPI score >2**	**0.007**			**0.016**		
**RDW >46.2 fL**	**0.01**	**1.895(1.063-3.380)**	**0.030**	**0.004**	**2.04(1.115-3.746)**	**0.021**

Abbreviations: ECOG PS, Eastern Cooperative Oncology Group performance status; HB, Hemoglobin; LDH, lactate dehydrogenase; EBER, EBV-encoded RNA; IPI, international prognostic index; KPI, Korean Prognostic Index; PINK, prognostic index of natural killer lymphoma; PFS, progression-free survival; OS, overall survival; Note: When the *P* value lower than 0.05, the corresponding factor was added into the multivariate analysis, and only the significant factors were listed for the results of multivariate analysis.

### Combining RDW to IPI, KPI, and PINK score improves survival prediction and risk stratification

**Figure 4:**
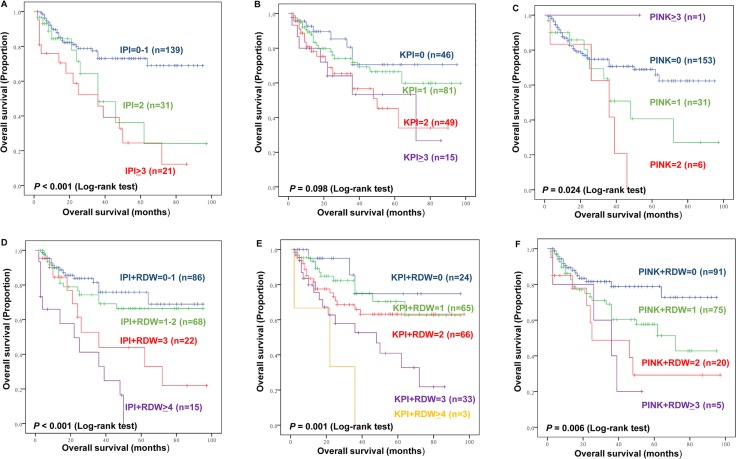
Kaplan–Meier plots of overall survival (OS) by International Prognostic Index (IPI) **(A)**, Korean Prognostic Index (KPI) **(B)**, Prognostic Index of Natural Killer lymphoma (PINK) **(C)**, Kaplan–Meier plots of OS by the new model combining RDW and IPI **(D)**, and KPI **(E)**, and PINK **(F)**.

We conducted a model for combining RDW to the IPI, KPI, and PINK. Briefly, patients with an elevated RDW level (RDW>46.2 fL) were allocated a score of 1, while patients without an elevated RDW level received a score of 0. We then added the RDW score to the IPI, KPI, and PINK scores to generate this new prognostic model. We performed C-index analysis to evaluate the discriminatory impact of RDW on OS. IPI and PINK scores were found to be significant with C-index analysis in OS (0.607 and 0.537, respectively) (Figure 4A, 4C; Table 4). No significance was found between KPI and OS with a C-index of 0.588 (Figure 4B; Table 4). After combining RDW with the IPI, KPI, and PINK scores, the new prognostic model showed significant association with OS, and survival prediction and risk stratification were improved as indicated by C-index (0.640, 0.639, and 0.603, respectively) (Figure 4D, 4F, 4E; Table 4).

**Table 4:** C-index for Discriminatory Values on Survival

	C-index for OS
**IPI**	0.607
**IPI+RDW**	0.640
**KPI**	0.588
**KPI+RDW**	0.639
**PINK**	0.537
**PINK+RDW**	0.603

**Figure 5:**
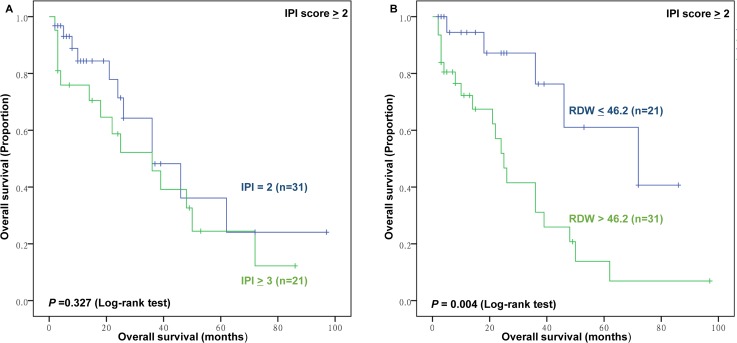
The prognostic value of high International Prognostic Index risk (IPI) and RDW in high IPI group **(A)** Overall survival (OS) of patients with IPI score ≥2 according to IPI; **(B)** OS of patients with high IPI (IPI, score ≥2) according to RDW ≤46.2 fL versus RDW >46.2 fL.

**Figure 6:**
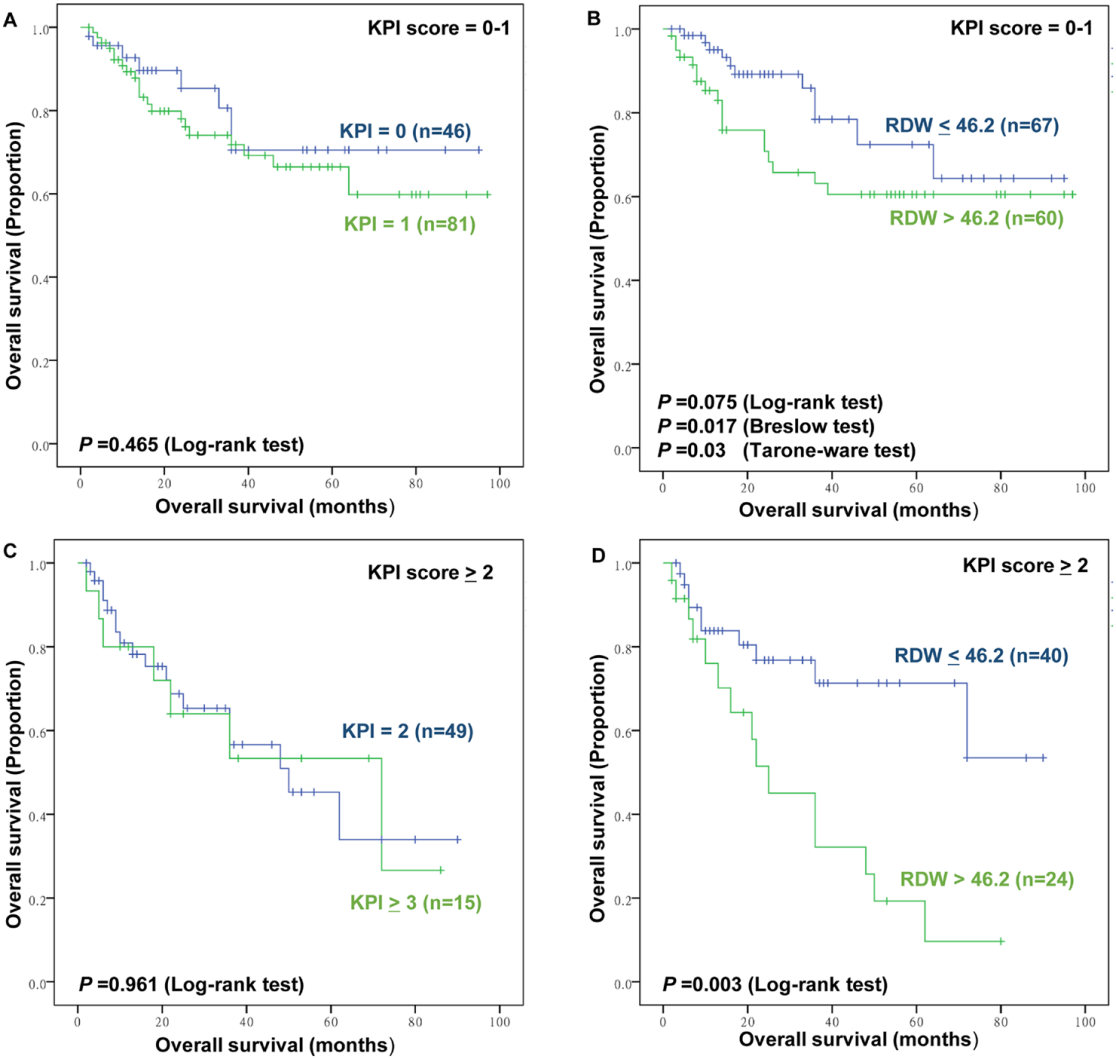
The prognostic value of Korean Prognostic Index (KPI) and RDW in low and high KPI group **(A)** Overall survival (OS) of patients with low KPI (KPI, score 0-1) according to KPI **(B)** OS of patients with low KPI (KPI, score 0-1) according to RDW ≤46.2 fL versus RDW >46.2 fL. **(C)** OS of patients with high KPI (KPI, score ≥2) according to KPI **(D)** OS of patients with high KPI (KPI, score ≥2) according to RDW ≤46.2 fL versus RDW >46.2 fL.

**Figure 7:**
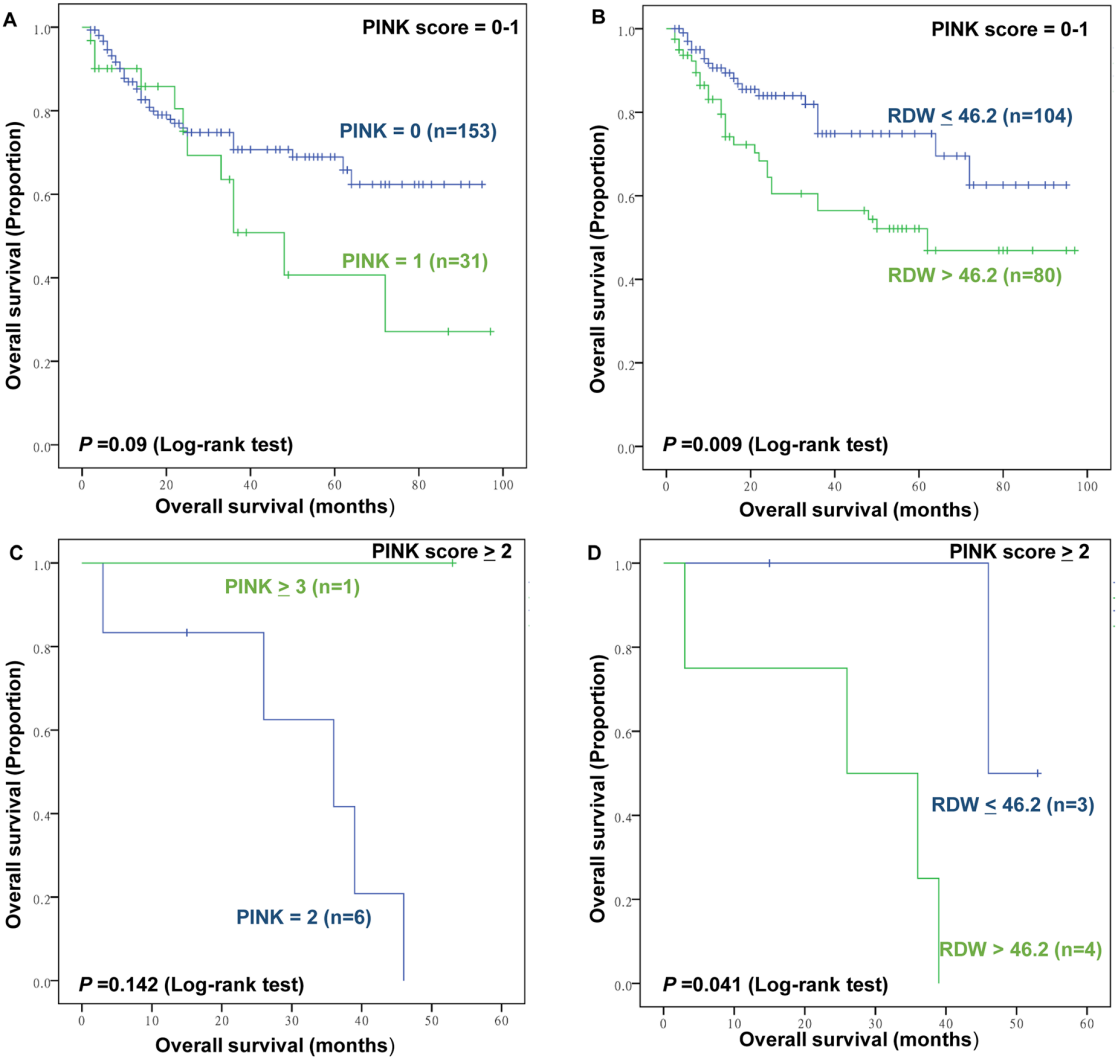
The prognostic value of Prognostic Index of Natural Killer lymphoma (PINK) and RDW in low and high PINK groups **(A)** Overall survival (OS) of patients with low PINK (PINK, score 0-1) according to PINK; **(B)** OS of patients with low PINK (PINK, score 0-1) according to RDW ≤46.2 fL versus RDW >46.2 fL; **(C)** OS of patients with high PINK (PINK, score ≥2) according to PINK (**D**) OS of patients with high PINK (KPI, score ≥2) according to RDW ≤46.2 fL versus RDW >46.2 fL.

Furthermore, subgroup analysis was conducted to further investigate the effect of RDW to IPI, KPI, and PINK score. The RDW failed to significant stratify patients with IPI scores 0–1 (*P*>0.05, data and figure not shown). However, the RDW distinguish patients with good outcomes from those with inferior outcomes among patients with IPI scores≥2 (Figure 5). For the KPI and PINK model, the RDW can stratify patients into a high-risk group or low-risk group (Figure 6; Figure 7).

**Figure 8:**
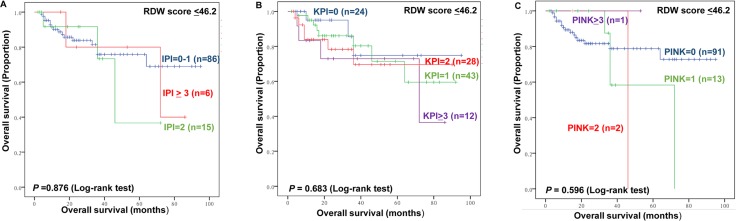
The impact of IPI **(A)**, KPI **(B)** and PINK **(C)** by RDW ≤46.2 fL.

Conversely, the IPI, KPI, and PINK prognostic models failed to show significant prognostic value among patients with RDW<46.2 fL (*P*=0.876, *P*=0.683, *P*=0.596, respectively, Figure 8).

## DISCUSSION

This retrospective study included 191 patients newly diagnosed with ENKTL to clarify the prognostic value of baseline RDW in pretreatment patients with ENKTL. Our results demonstrate that RDW is an independent prognostic factor for OS and PFS in patients with ENKTL given radiotherapy-based treatment. Moreover, combining RDW to IPI, KPI, and PINK scores can improve survival prediction and risk stratification. To our knowledge, this is the first report on the prognostic value of RDW in patients with ENKTL.

The RDW was used for the differential diagnosis of anemia for decades. However, in the recent years, RDW has gained notable renaissance. A variety of studies have found RDW to be a simple, robust, and convenient parameter associated with various human disorders such as cardiovascular disease [9], acute pulmonary embolism [10], diabetes [11], kidney disease [12, 13], liver disease [14], and chronic obstructive pulmonary disease (COPD)[15], but even more importantly, with overall mortality in the general population [3, 16, 17]. Recently, studies have demonstrated that RDW is prognostic factor for a variety of cancers [4]. Accumulating evidence has shown that RDW has important clinical significance. However, is RDW a risk factor or a simple epiphenomenon of an underlying biological or metabolic imbalance? Lippi et al. demonstrated that an elevated RDW involved both impaired erythropoiesis and abnormal erythrocyte metabolism and survival that mirrored a variety of abnormalities, such as shortening of telomeres length, oxidative stress, inflammation, erythrocyte fragmentation, poor nutritional status, hypertension, dyslipidemia and abnormality of erythropoietin function [3].

Although the IPI, KPI and PINK are important prognostic models in patients with ENKTL with specific treatments, they have not been previously evaluated in ENKTL with radiotherapy-based treatment. In this study, they show significant association with OS and PFS in univariate analysis, whereas they also have some disadvantages. A shared disadvantage is that more than 70% of the cases were classified into the low-risk group, which is consistent with previous studies [18, 19]. The RDW can effectively divide the low-risk patients into two groups, yet it failed to significantly stratify patients with IPI scores 0–1. Additional analysis found that the IPI, KPI and PINK were unable to clearly categorize high- and intermediate-risk groups. The high-risk group determined by IPI, KPI and PINK were further classified into two groups with significantly different survival outcomes with RDW. In order to investigate the impact of IPI, KPI, and PINK by RDW, these models were utilized to evaluate the RDW low-risk patients. All three models failed to significantly show significant prognostic value in patients with RDW≤46.2 fL. Finally, we constructed a new model by combining RDW with the IPI, KPI, and PINK scores. The new model showed more powerful prognostic value than the corresponding original models. Thus, we have determined RDW as a novel and powerful prognostic factor, which may be more useful than previous prognostic models. This study merits further prospective and multi-center studies to validate the prognostic value of RDW in ENKTL.

Inflammatory conditions are present before or after an oncogenic change occurs [20]. Cancer-related inflammation is regarded as a hallmark characteristic of cancer development and progression [21]. Inflammatory cells release various signaling molecules in the tumor microenvironment, which serve as effectors of tumor cell proliferation, survival and migration [22]. Meanwhile, inflammation associated markers are frequently investigated to develop cost-effective predictors for patients with cancer [23–26]. Lippi et al. found that RDW is robustly associated with two most widely used plasma inflammatory indices: circulating high-sensitivity C-reactive protein (hsCRP) and erythrocyte sedimentation rate (ESR) [27]. Additionally, in the healthy population and in patients with DLBCL, RDW was correlated with CRP [8, 28]. RDW is also associated with nutritional indicators, including albumin, iron, folate, and vitamin B12 in patients with cancer [8, 29]. Though the mechanism of RDW association with cancer patient survival is unclear, a possible explanation is that high levels of RDW mirror underlying inflammatory and malnutrition conditions which weaken erythrocyte maturation and causes inadequate production of the hormone erythropoietin, malnutrition, or oxidative damage. As such, the decreased survival seen in patients with elevated RDW may be a symptom of the underlying inflammation and malnutrition. Additional research is needed to explain the chemical and molecular mechanism relating RDW and cancer patient mortality.

According to National Comprehensive Cancer Network (NCCN) guidelines for Non-Hodgkin's lymphoma version 2.2015, the risk factors for ENKTL include age>60 years, ECOG PS score≥2, B symptoms, increased LDH, regional lymphadenopathies, local tumor invasion, histological expression of Ki-67, Epstein-Barr virus (EBV) DNA > 6.1E+7/ml, and IPI score >2. With the exception of EBV DNA, due to insufficient data, the rest of the risk factors were evaluated in our study. While half of these risk factors show significant association with survival in the univariate analysis, four factors (age>60 years, B symptoms, regional lymphadenopathies and Ki-67>50%) were not significantly prognostic for OS and PFS. Though age>60 years as a risk factor is investigated in many studies, some studies found that it was not predictive of survival [30–32]. Several previous studies also did not find significant associations between B symptoms and survival outcomes [33–35]. B symptoms was constructed in the pre-asparaginase era, and might lose its value in the era of asparaginase therapy. Regional lymphadenopathies was recognized as an independent risk indicator for worse OS in KPI [30]. Nevertheless, in our study, regional lymphadenopathies was not a significant predictor for OS, which is consistent with results presented by Kim et al. [36]. This difference might be related to better local control in these two studies, because regional lymph nodes might be included in the radiation field. The relationship between Ki-67 expression and outcome with lymphoma showed strong heterogeneity among various studies [37]. In Au et al.'s study overall survival did not differ significantly between patients with and without Ki-67>50% in univariate analysis [19]. We also found that AA III/IV shows no association with OS and PFS of patients with ENKTL, which is inconsistent with the results of other studies [30, 36]. This may result from the limited number of AA III/IV (7, 3.7%) in our study and differences in primary treatment. However, a second study has reported results consistent with ours [19]. Notably, due to some common limitations such as retrospective research, sample size imbalance between Ann-Arbor staging I/II and III/IV, uniform treatment (radiotherapy-based treatment), clinical heterogeneity for ENKTL, and the fact that some risk factors show negative results in our study, further well designed prospective studies are necessary.

In conclusion, this study demonstrates a dependent association between high RDW and poorer clinical outcomes in ENKTL patients treated with radiotherapy-based treatment. Further studies are warranted to confirm the prognostic value of RDW.

## MATERIALS AND METHODS

### Patient selection and data collection

We retrospectively enrolled 191 consecutive patients newly diagnosed with ENKTL in Sichuan Cancer Hospital between January 2008 and November 2016. All the patients included in this study were required to meet the following criteria: (1) pathologically and immunohistochemically confirmed diagnosis of ENKL, nasal type, by expert pathologists, according to World Health Organization (WHO) classifications [38], (2) available data regarding RDW at diagnosis, (3) sufficient clinical and follow-up data, (4) no acute infection or chronic active inflammatory disease, and (5) reception of radiotherapy-based treatment. This study was approved by the ethics committees of Sichuan cancer hospital. All patients consented to the use of their medical records for research purposes.

We collected the following pretreatment clinical and laboratory data: age, gender, physical examinations, Eastern Cooperative Oncology Group performance status (ECOG PS), B symptoms, serum lactate dehydrogenase (LDH), extranodal sites, Ki67, local invasiveness, regional lymphadenopathies, baseline RDW standard deviation (RDW-SD) levels, Ann Arbor (AA) stage, and comorbidities, including smoking, drinking, hemoglobin (HB) levels, hepatitis B (HBV), and other underlying diseases (including hypertension, hyperglycemia, hyperlipidemia and chronic bronchitis). International Prognostic Index (IPI) [39, 40], Korean Prognostic Index (KPI) [30], Prognostic Index of Natural Killer lymphoma (PINK) [36] for nasal NK/T-cell lymphoma were also used to perform survival analysis.

Regional lymphadenopathy was defined as the involvement of lymph nodes corresponding to N1-N3 of the primary lesion at the (Tumor, Node, Metastasis) TNM staging system. Local invasiveness was defined as T3 or T4 for upper aerodigestive tract NK/T-cell lymphoma (UNKTL) according to the 2002 TNM classification of the American Joint Committee on Cancer [30]. RBC analysis and RDW calculation was conducted on Mindray BC5800.

RDW was defined as the standard deviation (SD) of RBC volumes. Computed tomography (CT) or magnetic resonance (MR) of nasopharynx, neck, chest, and whole abdomen or positron emission tomography/CT (PET/CT) were utilized to evaluate patients’ disease.

### Treatment

The first-line chemotherapy regimens included non-anthracycline-based and anthracycline-containing regimens. The non-anthracycline-based regimens consisted of P-Gemox (pegaspargase, gemcitabine, and oxaliplatin), LVD (l-asparaginase, vincristine, and prednisone), and VDLP (etoposide, cisplatin, l-asparaginase, dexamethasone). The anthracycline–containing regimen mainly included CHOP (cyclophosphamide, doxorubicin, vincristine, and prednisolone). A total dose of 50–56 Gy was given to involved-field radiotherapy, with daily fractions of 2 Gy based on conventional fractionation (five fractions per week).

Patients received one of the following initial treatment modalities: (1) radiotherapy alone, (2) chemotherapy followed by radiotherapy, (3) concurrent chemoradiotherapy, (4) concurrent chemoradiotherapy followed by chemotherapy, (5) chemotherapy before and after radiotherapy, and (6) radiotherapy followed by chemotherapy. The treatment response was evaluated according to the standard response criteria for non-Hodgkin's lymphoma [41].

### Statistical analysis

All statistical analyses were conducted with IBM SPSS 20.0 (SPSS Inc., Chicago, IL), R software version 3.2.3, and Cutoff Finder. A web-based R software (Cutoff Finder, http://molpath.charite.de/cutoff) was used to determine the optimal cut-off value for RDW and HB [42]. Overall survival (OS) was defined as the period from clear diagnosis to death, lost follow-up or deadline. Progression free survival (PFS) was defined as the period from clear diagnosis of the tumor to first tumor progression, death, lost follow-up or deadline. Fisher's exact test was used to identify associations between categorical variables. The Kaplan-Meier method was used to investigate OS and PFS, and the log-rank test was used to evaluate the difference. When the *P* value of log-rank was close to 0.05, breslow test and tarone-ware test were utilized. When the *P* value was lower than 0.05, the corresponding factor was added into the multivariate analysis. Multivariate analysis was conducted by Cox proportional hazard model. Discrimination for survival data was investigated utilizing the C statistic with concordance index (C-index) [43, 44]. The C-index can evaluate the model's ability to classify individual patients into risk groups with different prognoses by estimating the probability of concordance between predicted and observed outcomes. C-index was calculated using Hmisc R package in R software version 3.2.3 [45]. A two tailed P value <0.05 was considered statistically significant.

## Abbreviations

RDW: red blood cell distribution width
HR: hazard ratio
CI: confidence interval
OS: overall survival
PFS: progression-free survival
IPI: International prognostic index
KPI: Korean Prognostic Index
PINK: prognostic index of natural killer lymphoma
ECOG PS: Eastern Cooperative Oncology Group performance status
LDH: lactate dehydrogenase
CHOP: cyclophosphamide + doxorubicin + vincristine + prednisone
P-Gemox: pegaspargase + gemcitabine + oxaliplatin
LVD: l-asparaginase + vincristine + prednisone
VDLP: etoposide + cisplatin + l-asparaginase + dexamethasone.
